# Credit Evaluation System Based on Blockchain for Multiple Stakeholders in the Food Supply Chain

**DOI:** 10.3390/ijerph15081627

**Published:** 2018-08-01

**Authors:** Dianhui Mao, Fan Wang, Zhihao Hao, Haisheng Li

**Affiliations:** Beijing Key Laboratory of Big Data Technology for Food Safety, School of Computer and Information Engineering, Beijing Technology and Business University, Beijing 100048, China; maodh@th.btbu.edu.cn (D.M.); hao_zhihao@126.com (Z.H.); lihsh@th.btbu.edu.cn (H.L.)

**Keywords:** blockchain, LSTM, credit evaluation system, food supply chain

## Abstract

The food supply chain is a complex system that involves a multitude of “stakeholders” such as farmers, production factories, distributors, retailers and consumers. “Information asymmetry” between stakeholders is one of the major factors that lead to food fraud. Some current researches have shown that applying blockchain can help ensure food safety. However, they tend to study the traceability of food but not its supervision. This paper provides a blockchain-based credit evaluation system to strengthen the effectiveness of supervision and management in the food supply chain. The system gathers credit evaluation text from traders by smart contracts on the blockchain. Then the gathered text is analyzed directly by a deep learning network named Long Short Term Memory (LSTM). Finally traders’ credit results are used as a reference for the supervision and management of regulators. By applying blockchain, traders can be held accountable for their actions in the process of transaction and credit evaluation. Regulators can gather more reliable, authentic and sufficient information about traders. The results of experiments show that adopting LSTM results in better performance than traditional machine learning methods such as Support Vector Machine (SVM) and Navie Bayes (NB) to analyze the credit evaluation text. The system provides a friendly interface for the convenience of users.

## 1. Introduction

As a global problem, food safety significantly affects the public health and food safety issues have been a major subject of numerous articles in the media. Over the past few years, many food scandal cases have been disclosed in the media such as the China milk scandal that emerged in 2008 and the UK horse meat scandal in 2013 [1]. Recently, the “gutter oil” scandal [2] has raised food safety fears once again in China. More and more food safety problems have arisen and caused illnesses that have gained national and government attention. These problems also expose the many cracks which exist in the current food safety management system.

In the food supply chain, there are multiple “stakeholders” [3] that act as key trading participants such as farmers, production factories, distributors, retailers and consumers. These traders prefer to selectively provide the food information that is beneficial to them in the trading process for making a high profit. This easily leads to food fraud and food safety problems. As with any hard-to-regulate field, regulators can hardly collect reliable and authentic information to implement supervision because of the unreliable information providers in the supply chain. In conclusion, because of the complexity of the food supply chain and “information asymmetry” [4] between traders, the credit risks of traders have increased rapidly. The credit of traders is a key factor affecting the food safety. Constructing a credit evaluation system based on the blockchain technology [5] could help the government regulators engaged in food safety obtain more reliable, authentic and sufficient information.

In recent years, some studies have linked blockchain technology with the food supply chain. For example, Tian discussed the potential benefit of blockchain technology in the agri-food supply chain. Then Tian proposed a traceability system based on blockchain to improve the transparency of the food supply chain and enhance food safety [6,7]. Nestle, IBM and Walmart have also conducted a study of blockchain [8] in order to manage supply chains with increased efficiency and to detect and mitigate against food safety problems. In October 2016, Walmart opened the Walmart Food Safety Collaboration in Beijing as it signed a collaboration agreement with IBM and Tsinghua University. It aims to implement food tracking in the supply chain by blockchain. However, there is still an existing gap in these researches in that they tend to focus on studies to explore traceability of food [9] rather than strengthen the supervision of traders. It has become clear that blockchain represents an opportunity to efficiently manage supply chain data across a complex network from farmers, production factories, distributors, retailers and consumers. Different from the direction of previous research, a credit evaluation system based on blockchain is presented in this paper. In the system, all transaction and credit evaluation of traders are grouped together and stored in blocks. They are logged and stamped with information about the time, amount and participants as if a notary were present at every transaction [10]. Based on blockchain, the system provides a transparent and accountable platform to supervise and mange traders in the food supply chain. It meets the requirements of traders for a better supply chain collaboration and supervised environment.

The challenges and contributions of this paper can be summarized in the following three points:

1) This paper implements a credit evaluation system that adopts blockchain technology to strengthen the supervision and management of traders in food supply chain. The whole flow of processing logic about the system is given by smart contracts which are written by “chaincode” [11].

Complexities in food supply chains and information asymmetry between traders lead to regulators hardly being able to gather reliable and authentic information to supervise and manage traders. Blockchain technology provides a feasible approach to solve these problems in the supply chain. Many studies have piloted the use of blockchain to trace items in supply chains [12]. However, blockchain technology is still in an early phase in the food supply chain, where researchers are much more focused on the traceability of food [13] rather than supervision and management of traders. Faced with this challenge, the system presented in this paper focuses on the regulation process in food supply chains. First, traders need evaluate trading partners by smart contracts after they conduct a transaction. Then the transaction information, especially credit evaluation information about traders are gathered by the system. The gathered credit evaluations are input into a trained LSTM model for analysis and processing. Finally the system generates credit evaluation results and feeds back the results to regulators for supervision and management.

2) The system applies Hyperledger blockchain [14] to meet the challenges of the different authentications and permissions needed for different roles (traders and regulators) in the food supply chain. It also ensures that traders can be held accountable for the credit evaluation process while traders’ (or evaluators’) real identities remain anonymous.

The Hyperlydger Fabric blockchain is a consortium blockchain that comprises peer to peer networks. On the one hand, the permitted P2P network structure ensures that traders’ identities won’t be exposed and the contents of their comments won’t be leaked. Thus, that is more suitable for traders to effectively implement credit evaluations. Regulators can also obtain more reliable information to implement the supervision. On the other hand, traders and regulators on Hyperledger Fabric platform can acquire different authorizations and permissions issued by a Certificate Authority (CA). That means regulators can conveniently acquire the highest authority to supervise and manage traders in the food supply chain.

3) The merge system is responsible for combining blockchain technology and a deep learning model LSTM [15]. It adopts a trained LSTM model to directly analyze and process the gathered credit evaluation text about traders. Finally the credit evaluation results of traders are generated and the results fed back to regulators.

It is difficult for existing credit evaluation systems [5,16] to really play a regulatory role for the multiple stakeholders in the food supply chain. These system are more suitable for e-trading between two types of stakeholders (customers and sellers) rather than multiple traders. The system in the paper relies on the LSTM deep learning method to analyze the sentiments of credit evaluation text directly and generate a credit evaluation result. Compared with most existing methods, LSTM provides a much higher accuracy rate than SVM and NB in the experiments. The system not only establishes traders’ credibility, but also focuses on the supervision of traders using the credit evaluation results.

This paper is organized as follows: in Section 2, the materials and methods of the system are illustrated. It introduces the design decision and workflow of the system by adopting blockchain technology and the LSTM model. Section 3 provides an introduction of the implementation and results of the system. The paper next focuses on the discussion of the system by introducing the related work about contribution of other researchers on this topic in Section 4. Section 5 presents the conclusions and future directions of efforts about the system.

## 2. Materials and Methods

**Figure 1:**
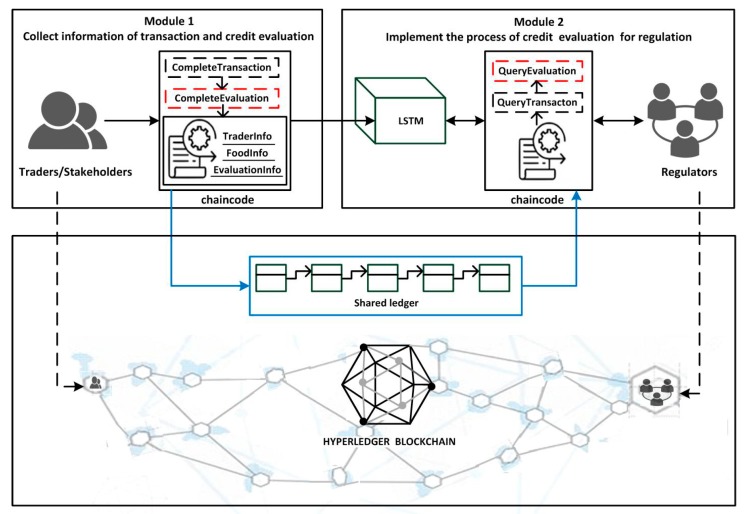
The architecture of the credit evaluation system.

This section shows the basic architecture of the credit evaluation system (see Figure 1 below) first.

The system composes of two different types of entities (Traders and Regulators) and consists of two modules (Module 1 and Module 2) based on the Hyperledger blockchain structure. For the purpose of understanding the system, the design decisions of systems that adopt the Hyperledger blockchain as underlying technology and the LSTM method are elaborated in the first subsection.

### 2.1. System Design Decision

#### 2.1.1. Hyperledger Blockchain

As Figure 1 shows, the system adopts the Hyperledger Fabric 1.0 framework as the underlying infrastructure. Hyperledger Fabric and Ethereum both are active platforms for distributed ledger solutions about blockchain. However these two popular frameworks have very different fields of application. Table 1 provides a brief summary of the two frameworks.

Compared with Ethereum, Hyperledger blockchain is a popular permissioned blockchain platform. P2P nodes in the Hyperledger blockchain form a consortium [13,14]. The system based on Hyperledger blockchain consists of multiple normal nodes representing traders and regulatory nodes. They acquire different authorizations and permissions issued by a Certificate Authority (CA). Registered users including traders and regulators generate ECerts using the CA. ECert contains one or more attribute names and values and specifies user’s name, roles, and passwords. Their authentication and permission information can be checked using “membersrvc.yaml” which is in the “membersrvc” folder. As shown in Figure 1, the system divides food supply chain users into two different types of identities named traders and regulators. To give further explanations, the nodes of traders and regulators have different features as follows:

**Traders.** Traders are the set of multi-stakeholders in the food supply chain such as farmers, production factories, distributors, retailers and consumers. Formally, they are the set of general nodes in permissioned P2P networks and they are treated equal during transaction. Their primary technical features are as follows:

- The nodes of traders mainly respond to block generation and credit evaluation generation when transactions are completed.
- Traders keep the records of transactions related to themselves.

**Regulators.** Regulators are the set of regulatory nodes. In permissioned P2P networks the set of regulator’s features are as follows:

- The nodes of regulators perform checking at regular intervals (for example, a week) from all nodes of traders to supervise and mange traders in the food supply chain.
- Regulators have the highest authority to manage the authentication, authorization and monitoring of traders. They can access and manage all information of traders in a blockchain.
- Regulators maintain the complete records of traders including the transaction and credit evaluation information.

The system based on blockchain has a lot of advantages compared to traditional systems. For example, the properties of distribution, detrusting, security, transparency and traceability have been used in the research of the food chain and food traceability. The rest of this subsection can be summarized and the feasibility studies about the credit evaluation system adopting Hyperledger blockchain pointed out:

1) Transparency and tamper-resistance. Blockchain consists of a continuously growing list of records, called blocks, which are linked and secured using cryptography. Each block typically contains a hash pointer as a link to a previous block, a timestamp and transaction data. All information of traders or transactions are stored in blocks. They are public to regulators and can hardly be modified. This can effectively avoid the risk arising from “information asymmetry” [17] in the supply chain.
2) Accountability and privacy. The credit evaluation system based on blockchain provides a reliable platform to collect information of transaction and credit evaluation. Traders’ identities are anonymous during the evaluation process. Thus they don’t have to worry about their identities being exposed or the contents of their comments leaked, which enhances users’ confidence. On the other hand, traders on the blockchain can be held accountable for their actions in the process of transaction or credit evaluation because of the transparent and tamper-resistant information provided.
3) Authorization and permission. The features of the consortium blockchain Hyperledger can provide different access control permissions via the CA. Regulators acquire higher authority than ordinary nodes of traders in the system based on Hyperledger blockchain, making it more suitable for regulators to supervise and manage traders in the food supply chain.
4) Chaincode. Hyperledger Fabric provides the logic of the system by smart contracts. Smart contracts run on the blockchain-based virtual machine and can be automatically executed by calling “chaincode”. Chaincode provides a variety of functions to invoke, update or query the data stored in the ledger. It can more quickly meet the needs of users and it is more effective for integrating regulators’ work into existing systems with a minimum of cost. In the system, traders complete the transaction and credit evaluation by chaincode. Regulators also call chaincode to query a transaction or gather the credit evaluation text of traders.

From the foregoing, Hyperledger Fabric as one of the most famous consortium blockchains provides a more flexible and easily used framework for the system to be developed. The above features are beneficial to establish the technological environment of a reliable credit evaluation system in the food industry.

#### 2.1.2. LSTM Method for Credit Evaluation

Sentiment analysis has long been a hot topic in natural language processing [18]. It can identify, extract and organize sentiments from generated texts such as product reviews. Many remarkable methods (SVM and NB) have been proposed for sentiment analysis. With the development of deep learning, many deep learning approaches (RNN and CNN) have emerged as powerful computational models improve the state-of-the-art in many sentiment analysis tasks [19]. The Long Short Term Memory network is a special kind of neural network architecture published in 1997 by Hochreiter and Schmidhuber, and later refined and popularized by many people in subsequent work. Compared with SVM and NB in the experiments, adopting the LSTM model to implement the sentiment classification of credit evaluation text acquires a much higher accuracy rate [18]. The next paragraphs describe existing problems of original credit evaluation systems and this paper presents the advantages by applying LSTM to solve these existing problems.

At present, major electronic trading platforms like “taobao.com” and “eachnet.com” have established credit evaluation systems to help regulators establish traders’ credibility [20]. These e-trading platform may be divide customers’ evaluations into two classes: “praise” and “bad review”, each corresponding to an integral evaluation. They use the accumulated credit rating model to add or subtract the original credit scores directly. For example, evaluation of successful transactions each corresponding to a credit score, specifically for the “praise” plus one point and “bad review” a one point deduction. The model is as follows:

R_n_ = R_n−1_ + r_n_, r_n_ ∈ {0, 1} (1)

In Equation (1), R_n_ and R_n−1_ respectively represent the credit score after the committed transactions numbered n and n−1, {0, 1} denotes the participants’ attitude {“negative”, “positive”}. Meanwhile, 1 is added or subtracted from the original credit scores. However, this model is too simple in that can’t take into consideration the credit rating of evaluators, the amount of transactions and so on. On this basis, “paipai.com” sets a weight for the amount of transactions It takes the product of the traders’ credit scores and the weight of the effective transactions’ amount as the final credit value for traders. The new evaluation model can be expressed as follows:

(2) $$R_{n}=\sum_{i=1}^{n}R_{i}\times W_{i},\;R_{i}\in \text{\{}0,\;1\text{\}};\;i,\;W_{i}\in \text{\{}0,\;+\infty \text{\}}$$

In Equation (2), R_n_ and R_i_ represent the credit score after the committed transactions number of n and i, respectively, W_i_ indicates the weight of the transaction N_i_’s amount. The weight of the transaction’s amount is determined in advance by the electronic trading platform. The greater the amount of transactions, the more objective and authentic the evaluations and the higher of the transactions’ weight. However, this model does not take into account the credit rating of traders. The credit evaluation is still not scientific.

In these exiting credit evaluation systems, customers directly give a credit evaluation of “praise” or “bad review” to sellers when the transaction is committed. Thus, these credit evaluation system just needs to focus on which class of e credit evaluation is given by costumers, then the class corresponds to a credit score. They don’t focus on the sentiment of the evaluation text. Meanwhile, there are only two types of stakeholders in the evaluation process. It is unilateral process for evaluating that sellers don’t have a chance to give credit evaluations to customers. It shows that existing systems are more focused on how to establish sellers’ credibility, making it difficult to play a regulatory role in the issue of trustworthy transactions and “information asymmetry” between “multi-stakeholders” in the food supply chain.

For these problems, this paper relies on LSTM to implement the evaluation process by analyzing the sentiment of credit evaluation texts (e.g., “The fruit doesn’t look very fresh” or “The logistics service is a little bit slower”) directly. The model focuses on supervision and management of traders by analyzing the credit evaluation text and generating at last a credit evaluation result. A two-way credit evaluation standard is adopted between traders. The system gathers traders’ transaction and credit evaluation information based on blockchain. Suppose a trader t ∈T has a credit evaluation from a trading partner during the transactions e ∈E. The model represents the credit evaluation text as a document d with n sentences {S_1_, S_2_, ⋯, S_n_}, where the ith sentence S_i_ consists of m words such as {w^i^_1_, w^i^_2_, ⋯, w^i^_m_}.

**Figure 2:**
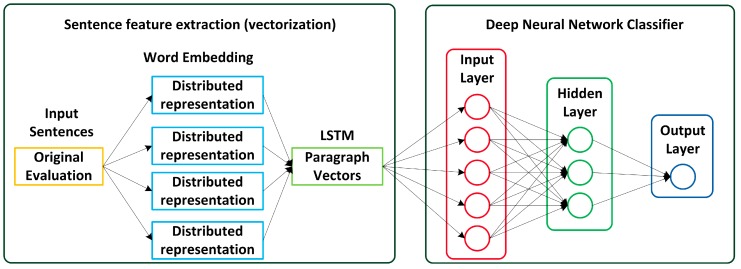
The architecture of LSTM.

The LSTM network is a type of recurrent neural network used in deep learning where very large architectures can be successfully trained. The architecture of LSTM network is shown in Figure 2.

An LSTM layer is composed of a number of “cells” that are explicitly designed to store information for a certain period of time. LSTM has many variations. In a simple LSTM one cell consists of three gates (input, forget, output). The mathematical theories of the LSTM cell in hidden layer are as follows:

(3) $$\left[\begin{array}{c} i_{j}^{i}\\ f_{j}^{i}\\ o_{j}^{i} \end{array}\right]=\left[\begin{array}{c} \sigma \\ \sigma \\ \sigma \end{array}\right]\left(W\cdot [{h^{i}}_{j-1},{w^{i}}_{j}]+b\right)$$

Each cell’s stored value is “guarded” by three gates that permit or deny modification of the cell’s value. i^i^_j_ is the feature matrix after output gate treatment. The “input” gate turns on when the input to the LSTM layer should influence the cell’s value. f^i^_j_ is the feature matrix after the forget gate selection. The “forget” gate and it turns on when the cell’s stored value should be reset. o^i^_j_ is the output feature matrix. The “output” gate and it turns on when the cell’s stored value should propagate to the next layer. In above formula, gates use a sigmoid activation, σ is the sigmoid function, W is the weight matrix of each gate, b is the offset of each gate. The parameters W and b need to be trained:

g^i^_j_ = tanh (W·[h^i^_j−1_, w^i^_j_] + b) (4)

In Equation (4), the input and cell state are often transformed with tanh. tanh is the hyperbolic tangent function. ⊙ represents the multiplication of matrix elements:

c^i^_j_ = f^i^_j_⊙c^i^_j−1 +_ i^i^_j_⊙ g^i^_j_ (5)

In this formula, the currently input cell state is determined by the previous and current feature matrix input. For example, when the input word w^i^_j_ is given, the current cell state c^i^_j_ and hidden state h_j_ can be updated with the previous cell state c^i^_j−1_ and hidden state h^i^_j−1_.

h^i^_j_ = o^i^_j_⊙tanh(c^i^_j_) (6)

In Equation (6), h^i^_j_ is the final output of the LSTM. It is determined by the o^i^_j_ of the output gate and the feature matrix input of the cell state at the current time calculated in Equation (5).

In above formula, [h^i^_1_, h^i^_2_, ⋯, h^i^_m_] stands for an average pooling layer to obtain the sentence representation of the credit evaluation text s_i_. When the input sentence of credit evaluation text S_i_ is given, the sentence embeddings [s_1_, s_2_, ⋯, s_n_] into LSTM and then we can obtain the document representation d through an average pooling layer just like the example of Equation (5).

LSTM supports time steps and it provides a way to address the time-series prediction problem. LSTM attaches great importance to content-based features rather than local text information. It is suitable for credit evaluation systems to predict the ratings of credit evaluations according to their text information directly. At present, the system just outputs the credit evaluation results in two ratings (“positive” and “negative”). For example, the results of “positive” means the trader obtains high praise and he is worthy of trust in the food supply chain, while a “negative” result means the trader has obtained many bad reviews and complaints in the food supply chain so he needs to improve the service quality.

### 2.2. Workflow of the Credit Evaluation System

Based on the two types of entities (traders and regulators) mentioned above, the credit evaluation system combining Hyperledger blockchain and LSTM consists of two specific modules as follows:

**Module 1. Collect transaction and credit evaluation information.** This module is designed primarily for the roles of traders. Traders can call the smart contracts which are written by “chaincode” to conduct the transactions through an interface of the application. When transaction is completed, traders evaluate the trading partner subjectively by smart contracts. During this process, the information of transaction and credit evaluation about the traders are all collected into the ledger.

**Module 2. Implement the process of credit evaluation for regulation.** Module 2 implements the credit evaluation and it is designed primarily for the roles of regulators. The credit evaluation texts about traders are created and stored inside the ledger by Module 1 during the trading process. Then regulators monitor periodically checking these information from ledgers. Regulators can directly access to the ledgers to search and gather the information of the traders by smart contracts on the blockchain. When regulators obtain the credit information text about traders, the module will utilise the trained LSTM model to analyze and process the sentiment of the gathered text. Finally, the system generates the credit evaluation result (“positive” or “negative”) by LSTM and feeds back the result to regulators. These pieces of information such as credit evaluation results during the process of evaluation are all stored into the ledger.

In the paper, credit evaluation-classification experiments with the binary-class LSTMs are performed and the model classifies any given credit evaluation results into the level “positive” and “negative”. “positive” and “negative” respectively represent the credit evaluation of “praise” and “bad review”. The regulatory agency in the food supply chain can verify and take corresponding measures in a timely manner depending on the credit evaluation result. Finally, this section presents a simple example to illustrate the work flow of the system in the following steps. Traders A and B are the subject of transaction in food supply chain:

1) Traders A and B complete a food trading transaction such as a sale of vegetables and fruit based on blockchain.
2) Trader B gives a credit evaluation to his trading partner A based on his satisfaction with his purchases, logistics service quality and food quality during the trade.
3) Collect the credit evaluation text of A at regular intervals (e.g., a week). These credit evaluation texts may be given by B, C and so on.
4) Input the gathered credit evaluation text into the trained LSTM model to analyze the sentiments of these texts.
5) The LSTM model outputs a credit evaluation result as “positive” or “negative”. For example, if A received more reviews like “The fruit doesn’t look very fresh”, “Its service is awful” or “The logistics service is a little bit slower”, then these texts are input into the trained LSTM model and the model will output a “negative” result for A.
6) The same steps 2 to 5 are performed by the other trader B.
7) Regulators periodically monitor and check traders’ credit evaluation results. They can verify the results and take corresponding measures in time.

## 3. Results

**Figure 3:**
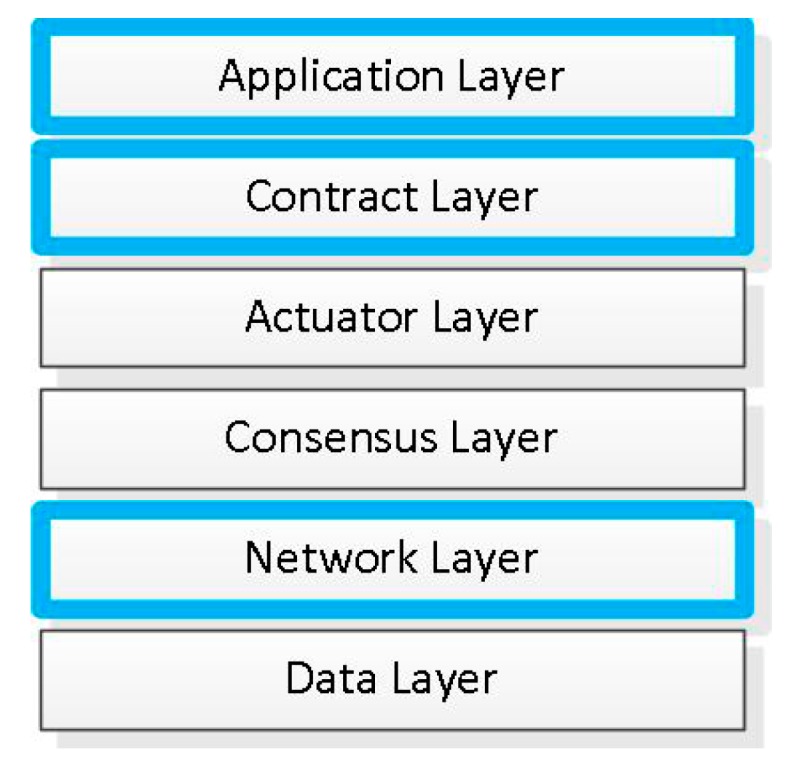
The architecture of blockchain.

As shown in Figure 3, blockchain decomposes into five separate layers (actuator layer is ignored in Hyperledger). The blue boxes in the graphic are the three layers we mainly pay attention to.

### 3.1. The Interface of the Integrated System and the Experimental Environment

To facilitate accessibility, the paper implements the prototype as a web application that can be accessed via an URI (Uniform Resource Identifier) from browser. Users can access the URI by manually running a local Hyperledger Fabric client.

**Figure 4:**
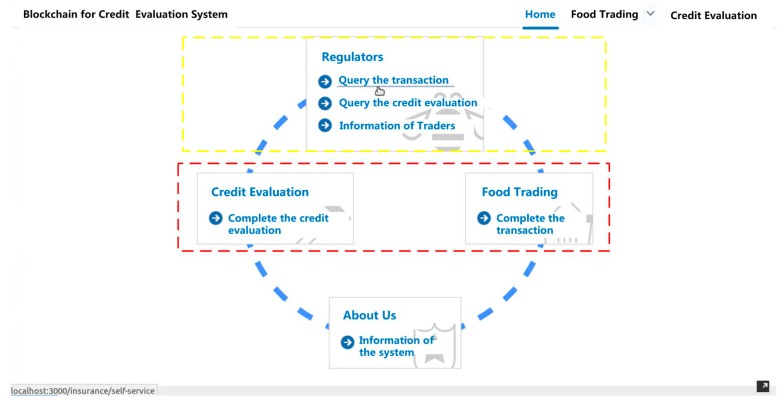
The interface of the integrated system for credit evaluation.

**Figure 5:**
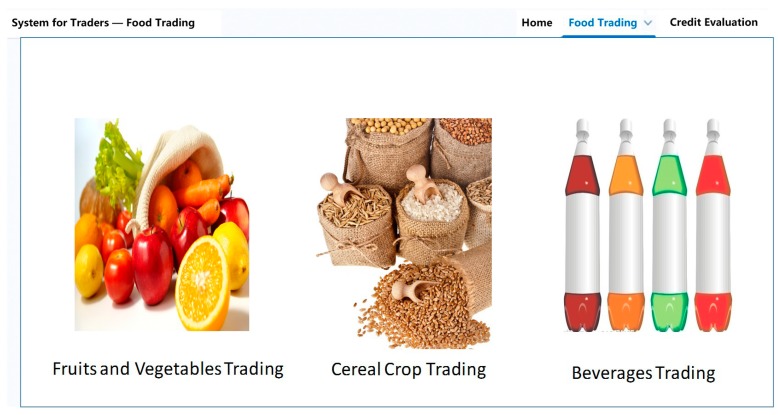
The interface for traders to complete the food transaction.

**Figure 6:**
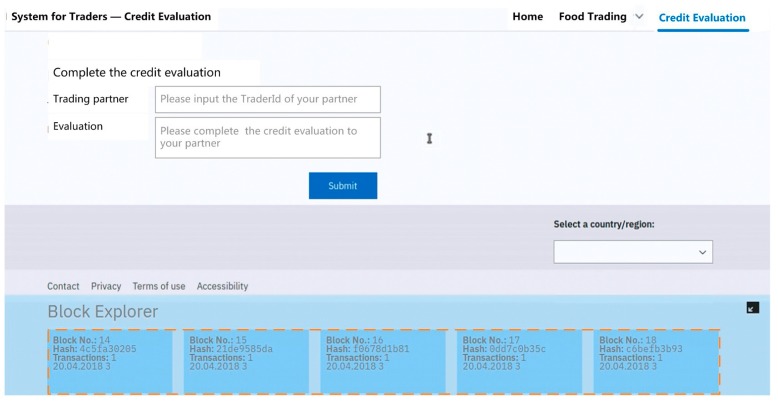
The interface for traders to complete the credit evaluation.

**Figure 7:**
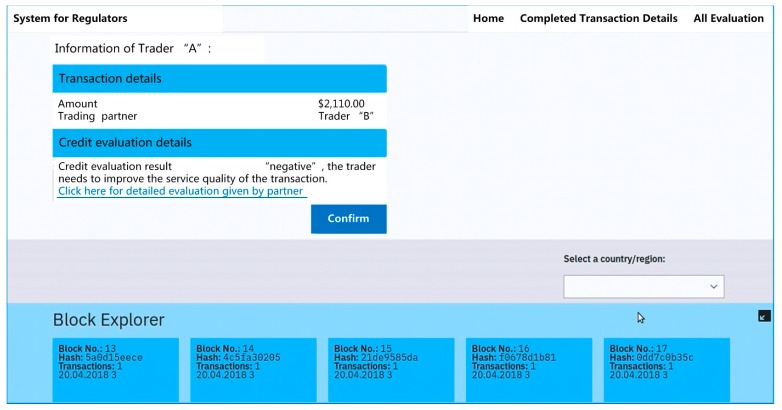
The interface for regulators to query the information.

As shown in the system architecture, the system consists of two basic components: Module 1: Collect information of transaction and credit evaluation and Module 2: Implement the process of credit evaluation for regulation. Figure 4 shows the detailed design of the integrated system for credit evaluation. The red box in Figure 4 below shows the functional interface of Module 1. It is mainly designed for traders. It provides the functions for traders to implement the food transaction (see Figure 5) and credit evaluation (see Figure 6) on the blockchain. The yellow box shows the interface of Module 2. It provides the functions for regulators to query traders’ information of transaction or credit evaluation (see Figure 7).

Figure 5 shows a snapshot of the web application for traders to complete the food transaction.

Figure 6 shows a snapshot of the web application for traders to complete the credit evaluation.

When traders complete the functions of Module 1, all information about the transaction and credit evaluation are grouped together in “blocks”. Blocks are some of the essential parts of the blockchain. As shown in the orange box in the figure, all information are logged and stamped with a timestamp and block hash. These technologies guarantee the data’s integrity and safeguard that it could not be tampered with. Thus all traders’ information can be stored in a secure and tamper-resistant manner on the blockchain. Figure 7 shows a snapshot of the web application for regulators to query trader’s (e.g., Trader A) detailed information about the transaction and credit evaluation.

All components and functions of the system above were developed in the Hyperledger Fabric version 1.0 environment. All the experiments about LSTM that we report in this paper were performed by Python 3.5. It adopts Keras as the structure. Keras is a deep learning library for Python, and it provides a large number of current popular deep learning models. More details of the basic environment are given in Table 2.

### 3.2. The Implementation of Smart Contracts

**Figure 8:**
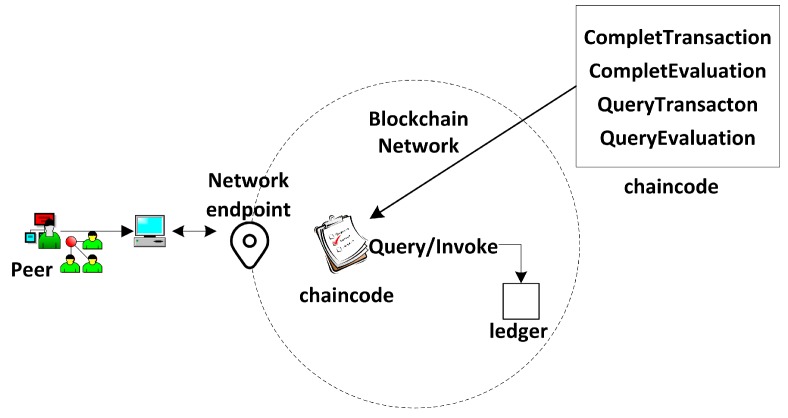
Trigger the chaincode and collect information of transaction and evaluation on the blockchain.

This paper has already mentioned the concepts of smart contracts and “chaincode” in the Hyperledger blockchain. Figure 8 shows the general flow of the smart contracts in Module 1 and Module 2. In the figure, chaincode is used to express the logic of the system flow. It describes the users’ need and provides code showing how the scenario was tackled, based on the architecture of the credit evaluation system. Chaincode is deployed in the Docker which is the container of the Hyperledger blockchain to perform the smart contracts. Once the chaincode is deployed, it obtains the unique address which users can use to interact with. Then its functions can be triggered by transactions sent to the smart contract address. Chaincode as a Turing-complete language is written flexibly in Go, Javascript and Java language. Considering that the majority of chaincode are written in Go, in this article we are only focusing on that one. Some snippets of chaincode are illustrated with the following example and these code implement the major functions of system. First of all, the chaincode needs to be initialized of the smart contracts both in Module 1 and Module 2. It also implements the initialization of two traders’ accounts (see Example 1).

**Example** **1.** A snippet of chaincode presenting the initialization process.

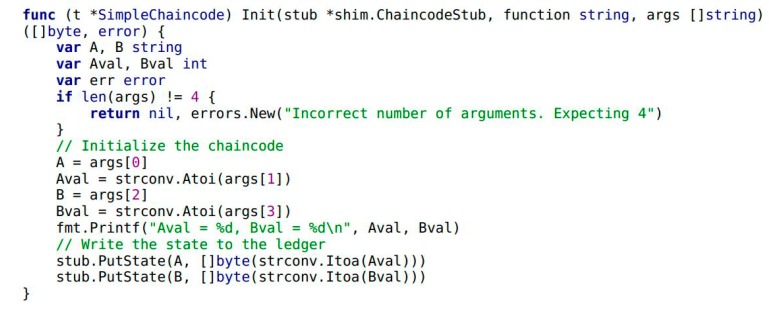

In Example 1, the variables of A and B are trader entities in the food supply chain. The variables Aval and Bval are the assets that A and B hold, respectively. Next, the transaction and credit evaluation information of the traders needs to be collected by Module 1 in Figure 1. It includes a smart contract in Module 1 and highlights the implementation by chaincode. Traders A and B complete the transaction by calling the internal function CompleteTransaction() of chaincode (see Example 3). When transaction is completed, traders B give credit evaluation to A by calling the function CompleteEvaluation() (see Example 4). As shown in Example 2, the function Invoke() encapsulates the functionality of CompleteTransaction() and CompleteEvaluation().

**Example** **2.** Snippet of chaincode providing a simple interface for use to implement the functions of Module 1.

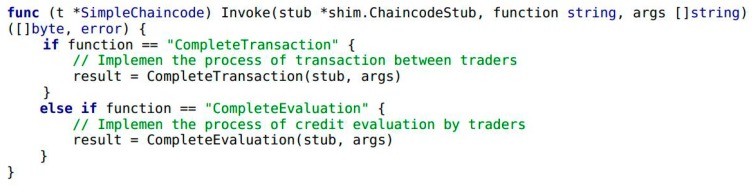

**Example** **3.** The following snippet of chaincode presenting the transaction process between traders A and B. The variable X is the value of the transaction. In the transaction, A transfers assets equivalent to X units to B.

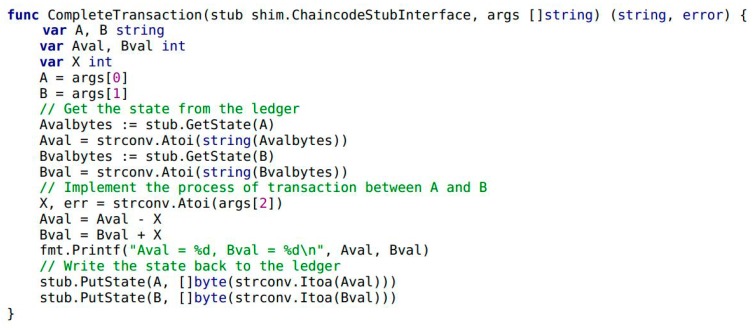

**Example** **4.** Snippet of chaincode presenting the credit evaluation process when a transaction is completed.

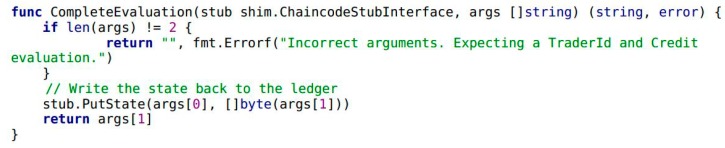

The credit system realizes the chaincode above mainly for gathering the credit evaluations from traders. Then the system implements the credit evaluation by Module 2 in Figure 1. It shows the process with two steps: (a) Acquire the gathered credit evaluation text by the function QueryEvaluation() of chaincode (see Example 6), (b) Input the gathered credit evaluation text into the trained LSTM model to analyze the sentiment of the text (this step will be described in detail in the next subsection). Finally, the output of the model shows the result of the traders’ credit evaluation. Regulators can request traders’ information by calling the function of Query() in the chaincode (see Example 5). It also contains two functions that are implemented by the chaincode of QueryTransaction() (see Example 7) and QueryEvaluation().

**Example** **5.** Snippet of chaincode providing a simple interface used to implement the functions of Module 2.

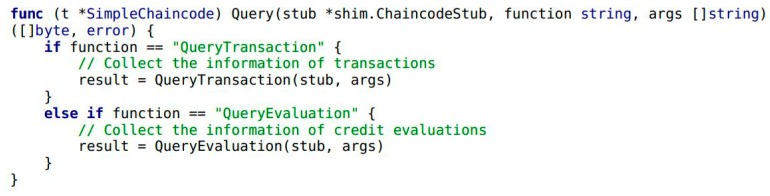

**Example** **6.** The following snippet of chaincode realizes the function of querying the transaction information of Trader A. TraderId is the unique identifier of the trader. The function queries the information of corresponding trader by accepting the variable of TraderId.

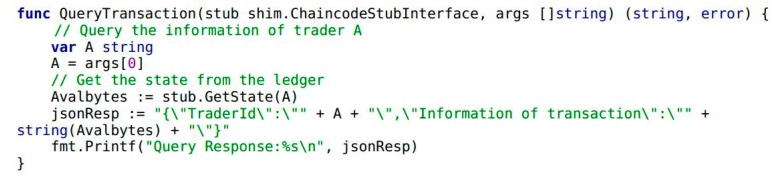

**Example** **7.** The following snippet of chaincode realizes the function of querying the credit evaluation information by TraderId.

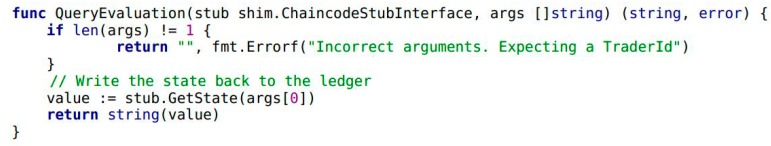

In the preceding examples, the main steps and related smart contracts to collect and generate the credit evaluation of traders are introduced. The following subsection focuses on the experiments and evaluation process of analyzing the sentiment of the gathered credit evaluation text by LSTM.

### 3.3. The Implementation of LSTM Model

**Figure 9:**
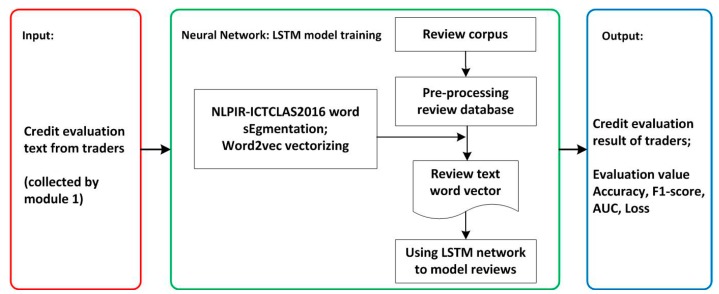
The general flowchart of the credit evaluation process by LSTM model.

The general flowchart of sentiment analysis process of credit evaluation text is illustrated in Figure 9. In this section, experiments are designed according to this flowchart to verify the performance of the sentiment analysis of Chinese credit evaluation text by the LSTM network.

#### 3.3.1. Experimental Dataset

For the purpose of the experiment, reviews of a Chinese text dataset (https://spaces.ac.cn/archives/3414) that were collected and shared by the nternet user named Jianlin Su are adopted. The crawler code is written in Python and the amount of the review corpus including the field “food” is more than 20,000 reviews.

In the experimental corpus, the review corpus may contain multiple features, such as in “The food stays fresh, and I like it very much, but the logistics service is not good”. In this paper, the experiments adopts the binary-class LSTM and the model classifies the given credit evaluation results into two levels: “positive” marked as 1 and “negative” marked as 0. Sentiment polarity of the food’s freshness is marked as 1, which indicates the traders hold a positive attitude about the food quality. Sentiment polarity of the logistics service feature is marked as 0, which represents the traders holds a negative attitude about the logistics service. Then the labeled corpus is randomly divided into training and validation (test) sets. The training set is used for the model training. The proportion of the quantity of the corpus in the training and validation set affects the experimental results during the process. In this experiment, we select “positive” and “negative” reviews with the same number from experiment review corpus. And we construct a training dataset and validation dataset of 7:3 based on the selected reviews data. At this point the pre-processing of the reviews dataset is complete.

Next, as shown in Figure 9, word segmentation for text of training dataset is performed using the open source word segmentation tool NLPIR-ICTCLAS2016, written by Zhang of the Chinese Academy of Sciences (Institute of Computing Technology, Chinese lexical analysis system, http://ictclas.nlpir.org/). The vectorization of the corpus is implemented by training word vectors through Google’s open source word2vec. After training and testing with the specimens, the model is able to analyze the credit evaluation collected by Module 1, and finally provides an objective evaluation to the traders.

#### 3.3.2. Evaluation Metrics

The model is tested using the validation dataset to verify the validation of the model and evaluate its performance. The results of sentiment analysis of the credit evaluation are evaluated by the experiment’s accuracy value, F1-score, Area Under the Curve (AUC) and loss.

**Accuracy.** Accuracy is the most intuitive performance measure. This metrics is used to evaluate how accurate the model’s prediction is compared to the true data.

(7) $$\mathrm{Accuracy}=\frac{\mathrm{TP}+\mathrm{TN}}{\mathrm{TP}+\mathrm{FP}+\mathrm{FN}+\mathrm{TN}}$$

**F1-score.** F1 score is the harmonic average of the precision and recall (in Equations (8) and (9)), where an F1 score reaches its best value at 1 and worst at 0. It takes both false positives and false negatives into account and F1 is usually more useful than accuracy:

(8) $$\mathrm{Precision}=\frac{\mathrm{TP}}{\mathrm{TP}+\mathrm{FP}}$$

(9) $$\mathrm{Recall}=\frac{\mathrm{TP}}{\mathrm{TP}+\mathrm{FN}}$$

(10) $$F1-\mathrm{Score}=\frac{2\times \left(\mathrm{Precision}\times \mathrm{Recall}\right)}{\mathrm{Precision}+\mathrm{Recall}}$$

In above formula, True Positives (TP) are the correctly predicted positive values which means that the value of actual class is yes and the value of predicted class is also yes. True Negatives (TN) are the correctly predicted negative values. False Positives (FP) mean that actual class is no and predicted class is yes. False Negatives (FN) mean that actual class is yes but predicted class in no.

**AUC.** AUC stands for area under the ROC curve. Receiver Operating Characteristic (ROC) curves typically feature true positive rate on the Y axis, and false positive rate on the X axis. ROC is actually slightly non-intuitive, while AUC can intuitively predict over accuracy for binary classification. The AUC value is equivalent to the probability that a randomly chosen positive example is ranked higher than a randomly chosen negative example and a higher value is better.

**Loss.** Training a network is finding the parameters that minimize a loss function (or cost function). Loss is often used in the training process to find the “best” parameter values for the model (e.g., weights in neural network). The cost function is the binary cross entropy. For a target G and a network output O, the binary cross entropy can defined as in Equation (11):

f(G,O) = −(G × log(O) + (1 − G) × log(1 − O))
(11)

Lower loss of the model at test time means experiment has lower prediction error and better performance. One can acquire the mean loss by feeding the model a batch of inputs.

#### 3.3.3. Evaluation Results of the Experiments

This section presents the results of the four experiments:

**Experiment** **1.** The following experiment tests the accuracy and loss of LSTM model on the corpus of Chinese text dataset with fixed amounts.

**Figure 10:**
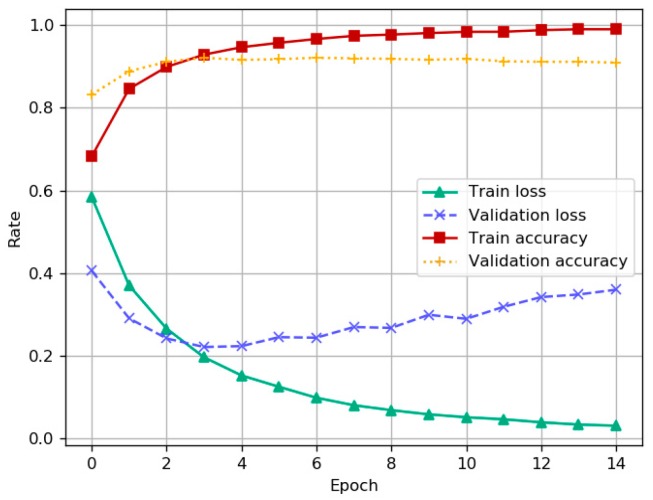
Performance test on LSTM model with the increase of experiment epochs.

The graph’s horizontal axis in Figure 10 shows the number of epochs to train the model. In this experiment, the value of epoch is 15. The vertical axis shows the value of accuracy and loss about training and validation data for every epoch in experiment.

**Experiment** **2.** The following experiment shows the fitting results and performance of different value of the epoch.

**Figure 11:**
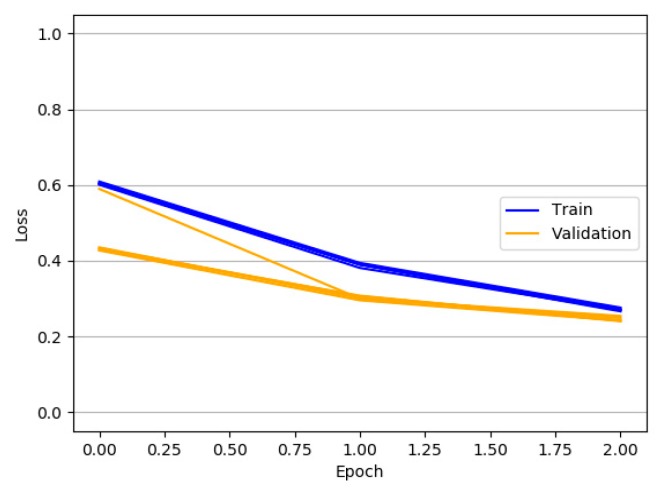
Curve fitting results on LSTM model in five experiments.

The corpus for training the LSTM is selected randomly, which means every time the performance evaluation result is different. Adjusting epochs with different value is necessary to find better fitting results and performance. This experiment performs five times and the performance is evaluated by the loss metric of the experiment. The training and validation trajectory is plotted in Figure 11. It shows that five experiments’ overall trend is roughly the same, and the curve fitting of test results performs well when the epoch value is set as 3.

**Experiment** **3.** This experiment tests the accuracy, loss and F1-score of the results on LSTM model.

**Figure 12:**
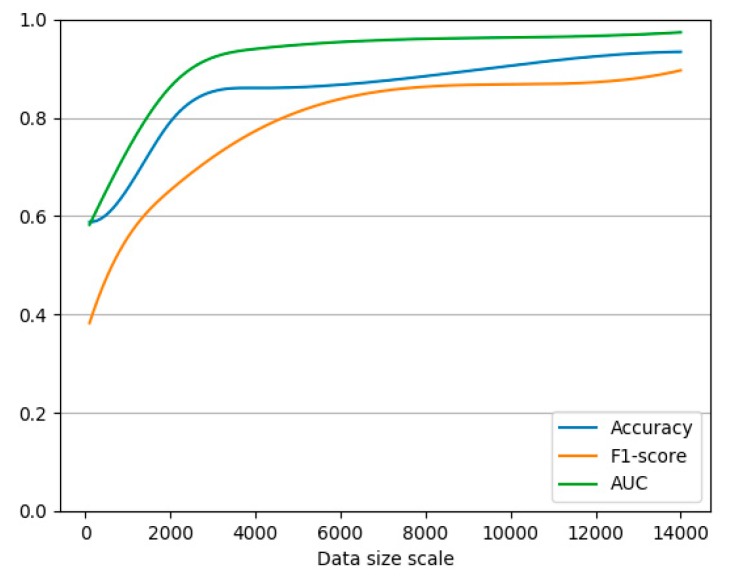
LSTM model performance changes with the scale of the corpus.

In this experiment, the performances of accuracy, loss and F1-score of the LSTM model become better than before with the increase of corpus and we set the epoch value as 3. The specific trend is shown in Figure 12: the graph’s horizontal axis shows the scale of both “positive” or “negative” corpus. The vertical axis shows the value of accuracy, loss and F1-score with the increase of data size. It shows that data accuracy and F1-score consistently increase with the scale of the corpus. Then it holds nearby a constant value of 90%.

**Figure 13:**
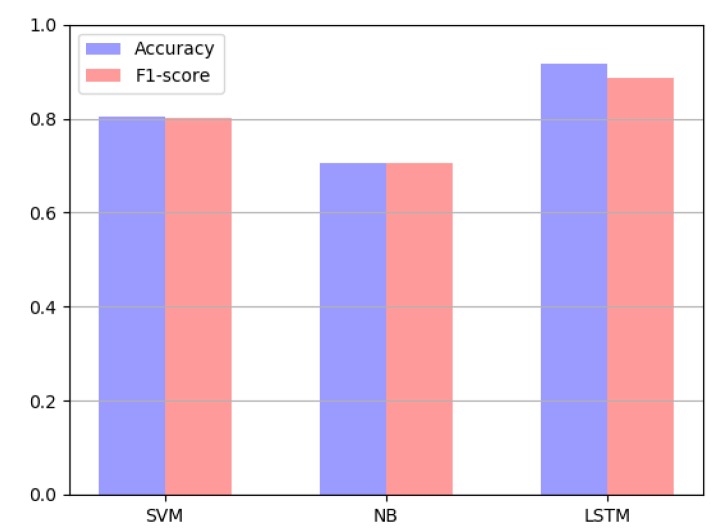
Comparison of results on the models SVM, NB and LSTM.

**Experiment** **4.** The final experiment results are compared the SVM model, NB model and LSTM model on the same Chinese text dataset as shown in Figure 13.

According to the data comparison in figure, the performance of the sentiment analysis of the LSTM model on the Chinese text dataset is greatly enhanced, compared with the traditional learning models SVM and NB.

In fact, many scholars have participated in completed experiments and published professional papers on the performance comparison different algorithms [21,22] about sentiment analysis. Compared to the SVM and NB models’ high computational complexity for classification the use of the LSTM networks for sentiment analysis of text features does not require a priori knowledge, such as syntactic parsing or a sentiment lexicon. LSTM can be more effective to learn the feature space and to capture temporal dependencies based on the recurrent neural network. LSTM model has long-term memory of the context of credit evaluation texts, which makes up for the disadvantage of the traditional sentiment analysis of neglecting credit evaluation feature text context, which makes the judgment of emotional tendency on credit evaluation features more accurate.

In further research, we will continue exploring the method of more exact and effective multiple classifications. Then the credit evaluations can be categorized into several grades such as grade 1, grade 0 and grade −1. For example, grade 1 represents that “The trader obtains high praise and he is worthy of trust in the food supply chain. The trader provides a perfect service in the process of trading and the food provided by the trader has good good-quality.”; grade 0 means that “The service quality of the trader is just common level and it is to be improved”; grade −1 represents that “The trader obtains many bad reviews and complaints in the food supply chain. The trader usually offers slow and rude service and he needs to improve the service quality.”

## 4. Discussion

The purpose of this section is to provide a brief overview of existing studies of blockchain technology and mainly focus on the studies which apply the blockchain technology in the food area. Then the credit evaluation system based on blockchain proposed in this paper is considered in comparison with the work of existing credit evaluation systems.

With the rapid development of computer technology, network technology and information technology, electronic commerce plays a more and more important role in modern people’s work and lives, and more and more traders prefer to make transactions on line. However, information asymmetry [4,17] between traders has led to a rapid increase of the credit risk of traders. With the higher demand for product quality and transparency in on-line transactions, credibility systems were created by the desires to effectively ensure the quality of transaction products. This is especially important in the area of food. Food is vital to people’s lives and food safety has always been valued by all sectors of society [1,2]. Regulators have realized that tackling modern technologies to build a credit evaluation system between traders is vital to promote food security during the transactions.

Many food safety assurance systems have been implemented in China to satisfy consumers’ demands for higher quality in the food market [23]. For example, foundation of China’s food safety assurance system named “Food Quality Safety Market Access System (QS System, China)” is implemented by AQSIQ in a compulsive way; Green Food certification, Organic food certification, China’s Brand-name Product, ISO quality system and other systems are implemented by producers in a voluntary way. However, they just point out the standards of food detection while they do not participate in the regulation process.

As mentioned above, in recent years, credit evaluation systems in the food area have been extensively studied by researchers. These credit evaluation systems can not only provide consumers with correct information, eliminate worries, and ensure their life and health, but also provide a decision-making reference for government departments or related regulators to manage food safety. For example, a quality credit classification evaluation index system of food enterprises [16] was put forward in 2015. It is composed of four key essentials: voluntary quality credit, ability, performance, and quality sustainability. It proposes a new quality credit evaluation methodology for food enterprises. Then researchers considered the credit risk in on-line transactions and proposed a system [5] to standardize the processes of online supply chain using big data and blockchain technology. This work provides a most vital step towards the system proposed in this paper.

As one of the best-known applications of blockchain, the crypto-currency Bitcoin was proposed in 2009 in the famous white paper named “Bitcoin: A Peer-to-Peer Electronic Cash System” by Nakamoto, which is speculated to be a fake name [24]. Nakamoto also proposed the blockchain protocols as a public, immutable and ordered ledger of records by combining a distributed database comprised of chronologically ordered and cryptographically interconnected blocks of transactions with a decentralized consensus mechanism and cryptographic security measures [10,11].

Before blockchain applications appeared, blockchain was regarded as the underlying technique support and the fundament of Bitcoin system. After that, many researchers abroad have began to analyse and mine the knowledge hidden behind crypto-currencies, such as Ethereum, Ripple [25], Litecoin [26] and so on. Additionally, several alternative blockchains have been proposed, such as sidechains [27]. These are all considered to be Blockchain 1.0 technologies. In combination with smart contracts, the technology has outgrown its origin in crypto-currencies. Then blockchain is known as a distributed ledger and the new technology of smart contracts [28,29] are considered Blockchain 2.0. In recent years, the idea of Blockchain 3.0 has been proposed to denote applications of the distributed ledger technologies. At first, it focused on the financial sector, such as the insurance claim process, the management of assets and the trading platform of E-commerce with its potential for disintermediation. Then, was applied to a variety of nonfinancial sectors like food.

According to a McKinsey study, blockchain is the core technology that now has the most potential to trigger the fifth wave of disruptive revolution after steam engines, electricity, information and Internet technology. With the gradual maturity of blockchain technology, many papers have proposed the application of blockchain to food [30]. Several companies and organizations are also exploring ways to use this shared, immutable ledger technology known as blockchain to “upgrade” the traditional food system. One of the most striking happenings occurred in Aug 2017 IBM [31] announced that it is working with a group of global food giants including Nestle and Walmart [8,32] to bring the benefit of blockchains to the food supply chain. Then they launched the blockchain food safety alliance in China with Fortune 500’s JD.com and Tsinghua University for the implementation of blockchain for tracking food in the supply chain. These applications [6,7,12] optimize business transactions and trading relationships with robustly secure business networks on blockchain both at scale and globally and aim to improve the confidence in food safety about the food trading. However, these researchers are more focused on the traceability of the traditional food supply chain [9,13] rather than supervision and management of traders in food supply chain. They are mainly devoted to promise the traceable and transparency for global food production. The exiting credit risk system proposed by Deng et al. [5] is applied to the finance supply chain and it is more suitable for trading between two parties [20]. The situation of multiple stakeholders (more parties) in the supply chain is not well considered either.

Different from these previous works, this paper aims at the problems existing in the supervision and management in the food supply chain especially “information asymmetry” [4,17] and “multple stakeholders” [3]. It provides an innovative system named “credit evaluation system based on blockchain for multiple stakeholders in the food supply chain” to implement the combination of blockchain technology and the food supply chain. Blockchain technology as a distributed ledger technology offers a decentralized, untrustworthy, accountable and transparent architecture that enhances the effectiveness of the regulation on the supply chain.

## 5. Conclusions

This paper provides a credit evaluation system based on blockchain technology and proves that the system proposed is effective for the food safety field. Blockchain ensures the authenticity of the information of transaction and credit evaluation about traders in the food supply chain. It resolves the situation with “asymmetric information” between “multiple stakeholders” because traders are held accountable for their actions during trading on the blockchain. The system also combines blockchain technology and the deep learning network LSTM to collect and analyze the credit evaluations of traders in the food supply chain. It strengthens the effectiveness of the supervision and management by generating and feeding back the credit evaluation result to regulators. Finally the goal of strengthening the food safeguards in the food supply chain is achieved.

By validating and analyzing the precision of the credit evaluation system by different experiments, we examine the performance of the evaluation metrics in the different models and with different indicators. The experimental results show that the credit evaluation system based on blockchain is feasible and efficacious on the Chinese text dataset about reviews by adopting the LSTM model. However, there are several limitations in this article. Firstly, we simply consider the emotion-tags of credit evaluation text into two level “positive” and “negative”. Thus, the method of more exact and effective multiple classifications of multi-class emotion-tags needs to be explored. Secondly, from the perspective of the dataset, the number of reviews in the text dataset in Chinese is not enough, which limits the representation of models to precisely analyze the sentiment of the credit evaluation. Future researchers need to train the model with more review text datasets. The performance of the experiments on an English dataset which is distinct from the Chinese dataset also needs to be considered in the future. Thirdly, there are still several major weaknesses in blockchain. For example, each node on the blockchain network needs to store the entire history of the blockchain and the growing blockchain size then becomes a growing concern issue. Leng et al. proposed a double chain architecture based on blockchain technology [33] to enhance the efficiency of blockchain in the agricultural supply chain, while because of blockchain’s relative novelty, this technology still has a long way to go.

## Figures and Tables

**Table 1:** Comparison blockchain platform of Hyperledger Fabric and Ethereum.

Characteristic	Hyperledger Fabric	Ethereum
Category	Consortium blockchain	Public blockchain
Description	Modular platform	Generic platform
Governance	Linux Foundation	Ethereum developers
Authority	Permissioned, private	Permissionless, public or private
Smart contracts	Chaincode (e.g., Go, Java)	Smart contract code (e.g., Solidity)

**Table 2:** Experiment environment.

Environment	Details
PC	Intel (R) Xeon (R) CPU 2.40 GHz (2 Processors), 12.0 GByte Memory
OS	Ubuntu 16.04 Desktop
Language	go 1.9.2 Linux/AMD64, jquery-3.1.1, bootstrap-3.3.7, Python 3.5
Containerization	Docker 17.10.0
Hash Function	SHA-256
